# A novel *PAX6* variant as the cause of aniridia in a Chinese patient with SRRRD

**DOI:** 10.1186/s12920-023-01620-w

**Published:** 2023-08-04

**Authors:** Qian Wang, Wen Bin Wei, Xiang Yu Shi, Wei Ning Rong

**Affiliations:** 1 Beijing Tongren Eye Center, Beijing Key Laboratory of Intraocular Tumor Diagnosis and Treatment, Beijing Ophthalmology & Visual Sciences Key Lab, Medical Artificial Intelligence Research and Verification Laboratory of the Ministry of Industry and Information Technology, Beijing Tongren Hospital, Capital Medical University, China. 1 Dong Jiao Min Xiang, Dong Cheng District, Beijing, 100730 China; 2 Ningxia Eye Hospital, People’s Hospital of Ningxia Hui Autonomous Region, Third Clinical Medical College of Ningxia Medical University, Huanghe Road, Jinfeng District, the Ningxia Hui Autonomous Region, Yinchuan, 750002 China

**Keywords:** Aniridia, Spontaneous reattachment rhegmatogenous retinal detachment, *PAX6*

## Abstract

**Background:**

The genotype characteristics and their associated clinical phenotypes in patients with aniridia were analyzed to explore pathogenic variants using whole-exome sequencing.

**Methods:**

One patient with aniridia was enrolled at the Beijing Tongren Hospital. Comprehensive ophthalmic and general examinations were performed on the patient. DNA was extracted from the patient, and whole-exome sequencing was performed to identify the causative variant. The pathogenicity of the variant was predicted using in silico analysis and evaluated according to American College of Medical Genetics and Genomics guidelines. Relationships between genetic variants and clinical features were analyzed.

**Results:**

In addition to the classical aniridia phenotype showing complete iris aplasia, foveal hypoplasia, and ectopic lentis, the patient also exhibited spontaneous reattachment rhegmatogenous retinal detachment (SRRRD). Whole-exome sequencing identified a novel heterozygous variant, exon8:c.640_646del:p.R214Pfs*28.

**Conclusions:**

The present study broadens the range of genetic variants described in aniridia and presents an aniridia patient with SRRRD.

## Background

Aniridia (OMIM, 106,210), a rare congenital panocular disease characterized by a variable degree of iris hypoplasia, affects roughly 1/64,000 to 1/96,000 live births without regard to sex or racial differences [1–3]. Aniridia can occur as an isolated ocular abnormality or as a manifestation of Wilms tumor-aniridia-genital anomalies-retardation (WAGR) syndrome or other related syndromes [3, 4]. Individuals with aniridia exhibit impaired visual acuity, nystagmus, and foveal hypoplasia [3]. Milder forms of aniridia result in better vision, subtle iris architecture changes, and normal foveal structures [5]. In addition to the prominent ocular features, aniridia is associated with a wide range of other abnormalities, such as sensory, neural, cognitive [6] and pancreatic involvement [7, 8]. A total of two-thirds of aniridia cases are autosomal dominant, have complete penetrance, and exhibit varied expression, whereas the remaining cases are sporadic [9–11]. Most cases of congenital aniridia cases are caused by pathogenic mutations in the *PAX6* gene (OMIM, 607,108) [12–14]. Rearrangements of*PAX6* neighboring regions, is considered to be the underlying pathogenic mechanism in a small subset of aniridia, and this phenomenon is known as the “position effect” [15]. Although, the phenotype varies between and within families; affected individuals usually show little variation between the eyes [3].

In Chinese aniridia patients, 96.9% of the causative sequencing changes are located in the*PAX6* gene [1], which is similar to the previous reports from two large cohort studies in different ethnic populations [16, 17]. Almost all of the mutations of *PAX6* have been identified in Chinese patients [1], and no hotspot regions have been reported. You et al. observed some genotype-phenotype correlations in a large cohort of Chinese patients with aniridia [1]. Intragenic mutations in *PAX6*inducing nonsense-mediated decay (NMD) or large deletions involving *PAX6*were detected in 96% of the patients with typical aniridia. In contrast, the majority of patients (68%) with milder forms of aniridia have run-on, missense, or splicing mutations unrelated to the NMD process [1].

In this manuscript, we describe a novel *PAX6* variant in a Chinese patient who was first diagnosed with retinal detachment and ectopia lentis, to broaden the genetic variant spectrum of this rare condition.

## Methods

The patient was recruited in accordance with the principles of the Declaration of Helsinki. The study protocol was approved by the Medical Ethics Committee of the Beijing Tongren Hospital and written informed consent was obtained from the patient for participation in this study and the publication of the results. Unfortunately, the patient was an orphan, had lost his parents since childhood, and had no siblings. Therefore, clinical information and blood samples from his parents were not available. The patient’s deaf-mute mother died in a traffic accident when he was 14 years old. The patient’s father had abandoned the family before the patient was born and has never been heard since. However, he was described by fellow villagers as having poor eyesight.

### Clinical examinations

Ophthalmological examinations included the measurement of best-corrected visual acuity, tonometry, and slit-lamp-assisted biomicroscopy of the anterior segment of the eye. Ocular ultrasound (MyLab 90, Esaote, Genova, Italy) was used to determine axial lengths and observe vitreous and retina. Using a non-mydriatic fundus camera (CR6-45 NM; Canon Inc., Tokyo, Japan), 45° fundus and anterior segment photographs were acquired. Wide-field photography was performed using non-mydriatic ultra-widefield imaging (Optos, Dunfermline, UK). Spectral-domain optical coherence tomography (Heidelberg Engineering Co., Heidelberg, Germany) was used to evaluate the macular structure. The patient also underwent cranial MRI, routine liver, kidney, and glycosylated hemoglobin examinations to rule out related systemic diseases.

### Karyotype analysis

Karyotype analysis on peripheral blood lymphocytes using conventional G-band by Trypsin using Giemsa (GTG banding) was performed.

### Whole exome sequencing

Peripheral venous blood sample (5 ml) was collected from the participant for genomic DNA extraction using a QIAmp DNA Mini Blood Kit (Qiagen, Hilden, Germany). Whole exome sequencing was performed on the probands. The exome was captured using the Agilent SureSelect Exon Capture Kit according to manufacturer’s instructions. Sequencing was performed using a high-throughput sequencer (Illumina, HiSeq XTen). Raw sequencing data were processed using Illumina basecalling Software 1.7 analysis software following which it was compared to the NCBI human genomic DNA reference sequence (NCBI build 37.1). To obtain all variants occurring in the DNA sequences of the samples, single-nucleotide variants (SNV) and insertion and deletion variants (Indel) were analyzed using SOAP software (http://soap.genomics.org.cn), and BWA software (http://bio-bwa.sourceforge.net), respectively. The BWA software was used to compare with the hg19 human genome reference sequence provided by UCSC, and the SNV and InDel variants were found out through GATK’s HaplotypeCaller, and then through professional database. Sanger validation was used to exclude false positives for potential pathogenic variants.

### In silico analysis

The pathogenicity of the variant was assessed for genetic variation according to the Standards and Guidelines for Interpretation of Sequence Variants published by the American College of Medical Genetics and Genomics in 2015. The variant sites were filtered and screened by integrating them into the normal human database, which includes the normal population gene frequency 1,000 genomes (1,000 genomes), EXAC (The Exome Aggregation Consortium), and EXAC-EAS (approximately 4,000 East Asians data under EXAC). An Minor Allele Frequency (MAF) < 0.005 was used as the criterion to exclude benign variants. The gnomAD (all_gnomAD and eas_gnomAD), were use to analysis the frequency of variants in the normal population (and the normal East Asian population) of the gnomAD database. When all predictions were pathogenic, the variants were classified as potentially pathogenic in combination with further evidences. Frameshift and nonsense variants including variants with experimental evidences of causing loss of protein function were classified as pathogenic variants. The online analysis tool Multalin (http://sacs.ucsf.edu/cgi-bin/multalin.py) was used for conservativeness analysis of variant loci [18].

## Results

### Clinical evaluation

**Fig. 1:**
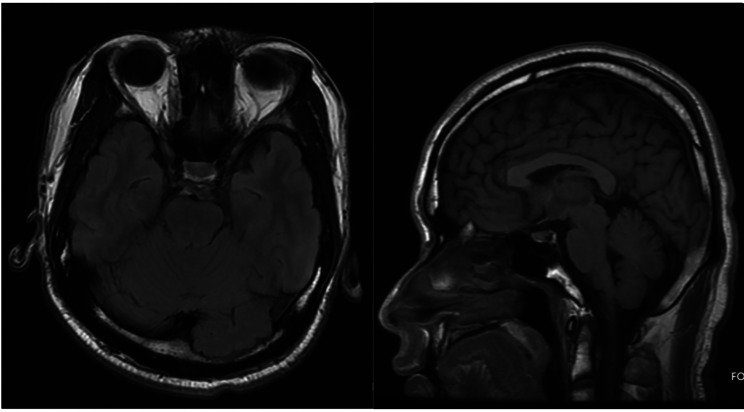
Brain MRI of the patient. Brain MRI showed no obvious abnormalities

A 20-year-old Chinese male patient with poor visual acuity in both of his eyes since childhood but had never sought medical attention was recruited for this study. The patient visited a local hospital more than three years ago (February 2019) due to decreased vision in the left eye and was diagnosed with ectopia lentis, aniridia, vitreous opacity in both eyes, and retinal detachment in the right eye. Lensectomy and vitrectomy were performed on the left eye. The patient’s general health and past medical history were unremarkable, and there were no obvious signs of WAGR syndrome. The patient’s development was essentially normal, and neither genital or behavioral abnormalities were present. Routine examinations of the liver and kidney, glycosylated hemoglobin levels (5.20%), and brain MRI revealed no evident abnormalities (Fig. 1).

**Fig. 2:**
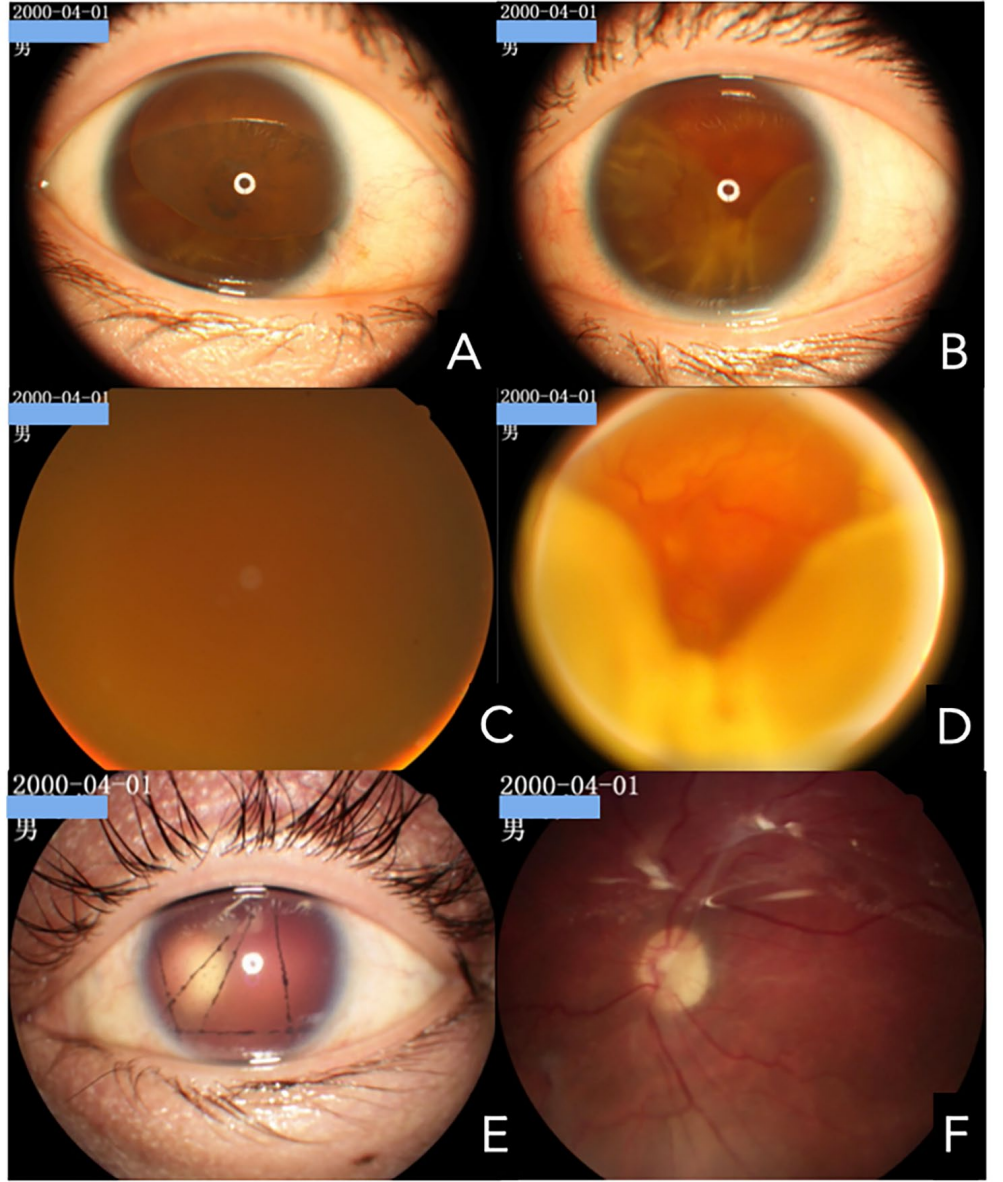
Fundus and anterior segment images of eyes using fundus camera.(**A**-**D**)Images of both the anterior segment and fundus of the patient during his initial visit to our hospital. (**E**-**F**) Images of the left anterior segment and fundus after the first operation on the left eye

**Fig. 3:**
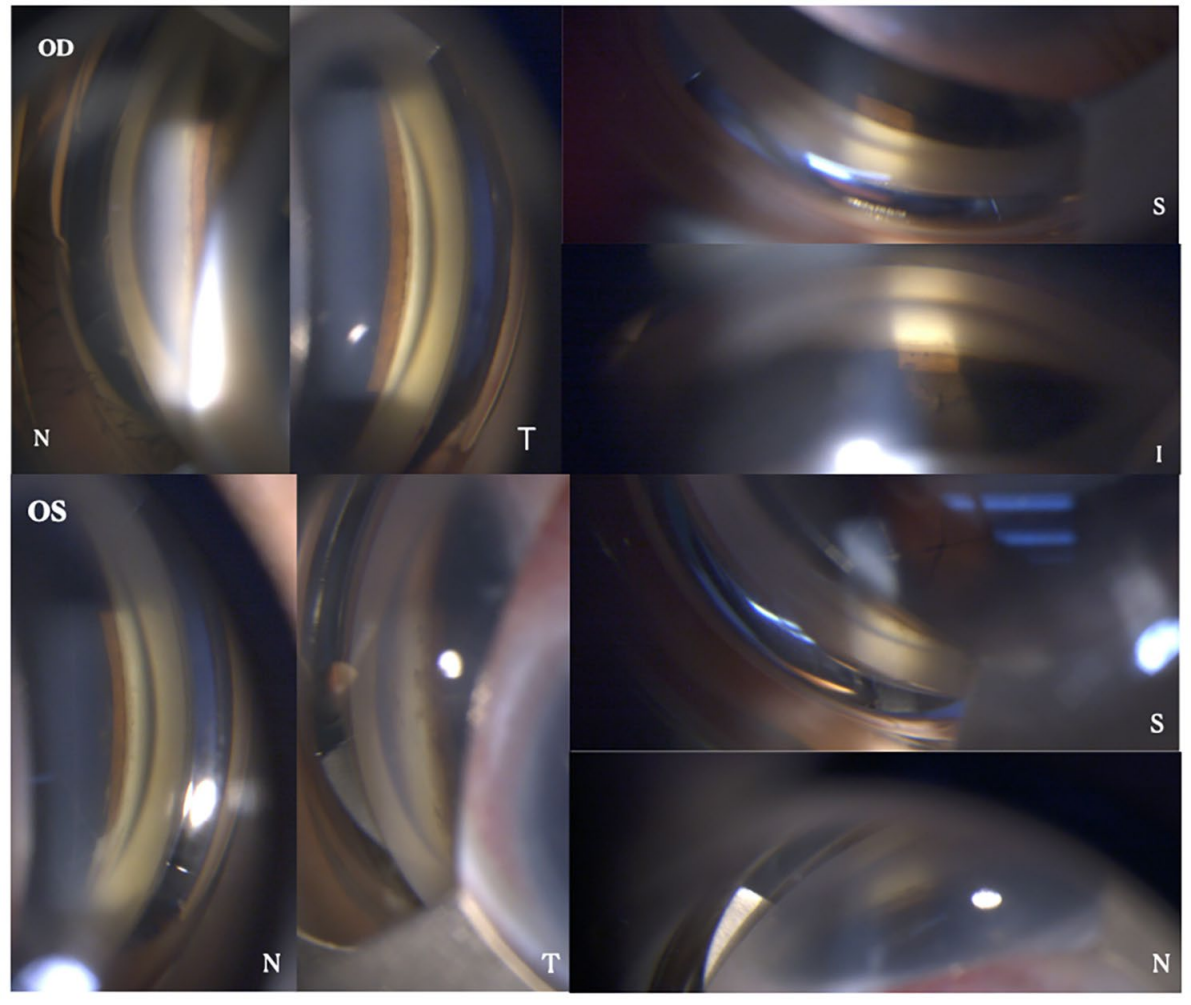
A gonioscopy showing normal chamber angles in both eyes

**Fig. 4:**
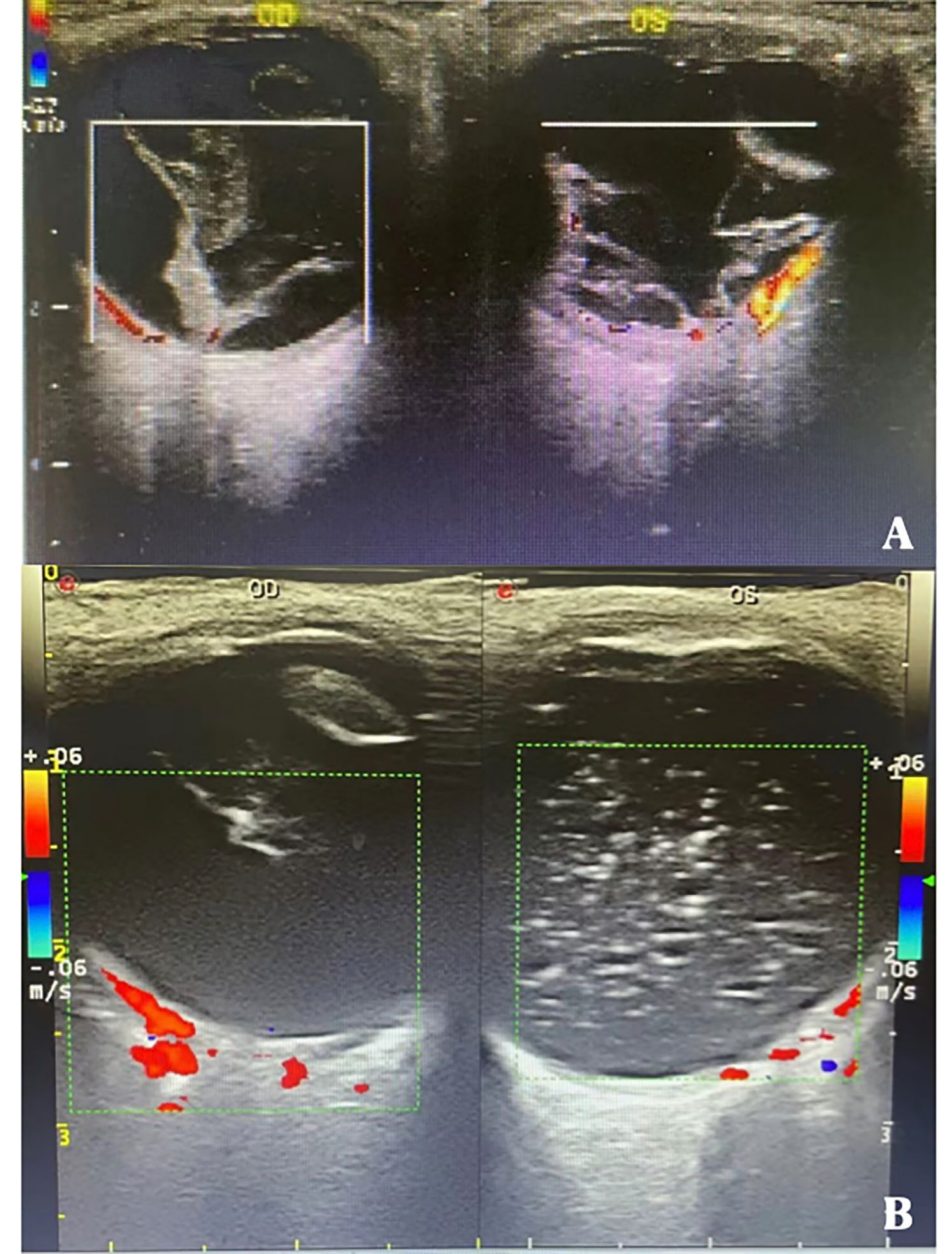
Ocular ultrasound of the patient. (**A**) During the patient’s initial visit to our hospital, binocular ultrasonography showed vitreous opacity and retinal detachment in both eyes. (**B**) Four months after the removal of the silicone oil in the left eye, ocular ultrasonography showed residual silicone oil in the left eye, and the retina was in place, while the retinal detachment in the right eye was spontaneously reattached without any surgery

Two months later (April 2019), the patient returned to our hospital with aggravated visual loss in the left eye. When admitted to the hospital, his best-corrected visual acuity was hand movements in both eyes. The intraocular pressure (IOP) measured by non-contact tonometry was lower than normal (7 mmHg in the right eye and 6 mmHg in the left eye). Anterior segment examination revealed total aniridia and nasal-superiorly subluxated lenses with cataracts in the right eye, and total aniridia and aphakic lenses in the left eye(Figures 2 A-B). There were no explicit abnormalities in the corneas of either eye, except for a small diameter (11 mm in both eyes). Gonioscopy revealed that the chamber angles in both eyes were approximately normal (Fig. 3). Fundus examination showed vitreous opacity and total retinal detachment with proliferative vitreoretinopathy in both eyes (retinal detachment in the right eye was only detectable under indirect ophthalmoscopy due to opacity of the refractive media) (Fig. 2 C-D). Ocular ultrasonography confirmed retinal detachment in both eyes (Fig. 4A). The patient underwent vitrectomy of the left eye combined with silicone oil tamponade.

Shortly after the surgery, the IOP in both eyes increased and returned to normal after treatment with Perithiazine and Alphagen. Three months following the surgery, visual acuity in the left eye improved to 10/200, the retina reattached, and the IOP was elevated to 31 mmHg (Fig. 2E-F). Except for the elevated IOP (39 mmHg) in the right eye, the remaining symptoms did not significantly change from three months prior. Silicone oil removal combined with endoscopic cyclophotocoagulation was performed in the left eye. Postoperatively, the IOP of the left eye returned to normal, and with the addition of tafoprostaglandins, the IOP of the right eye likewise returned to normal.

After the left eye had made a better recovery (30/200) four months later (October 2019), surgery was performed in the right eye. The visual acuity and anterior segment of the right eye did not change significantly compared with the initial visit; however, with the exception of vitreous opacity, indirect ophthalmoscopy did not reveal definite retinal detachment, which was further confirmed by ocular ultrasonography (Fig. 4B). During surgery, following the removal of the cloudy and dislocated lens and the cloudy vitreous, the fundus was examined in detail. No retinal detachment was found except for a suspicious retinal tear behind the ora serrata at the 11 o’clock position, and retinal photocoagulation was performed.

**Fig. 5:**
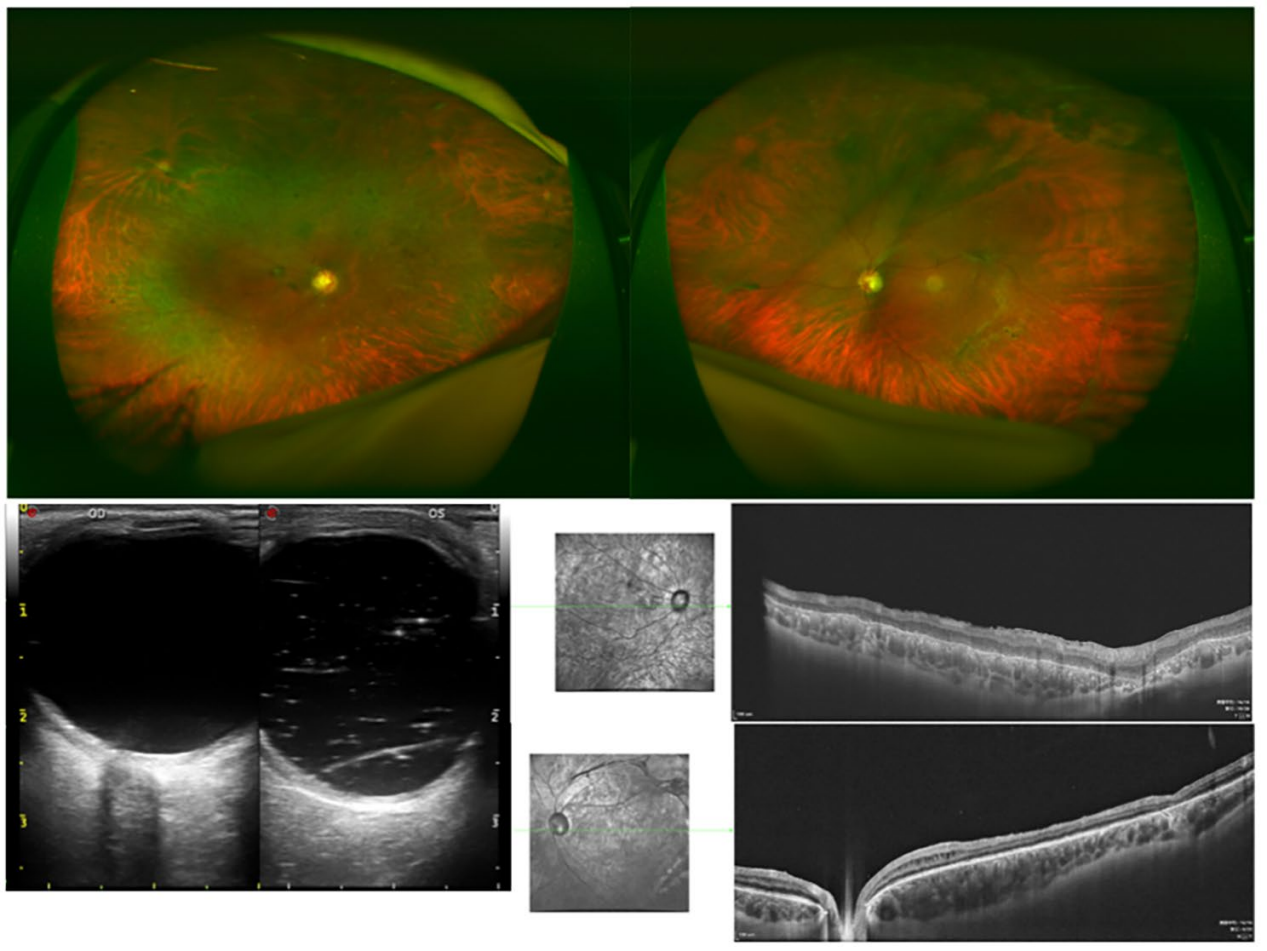
Examinations results during the final visit. The retina was in place in both eyes, and the macular dysplasia was identified. Optical coherence tomography (OCT) demonstrated the absence of foveal depression and extensive retinal atrophy in both eyes

During the final visit (December 2022), visual acuity was light perception in the right eye and 40/200 in the left eye. The retinas were in place in both eyes, and macular dysplasia was noted. OCT revealed the absence of foveal depression and extensive retinal atrophy in both eyes (Fig. 5).

### Mutation analysis

**Fig. 6:**
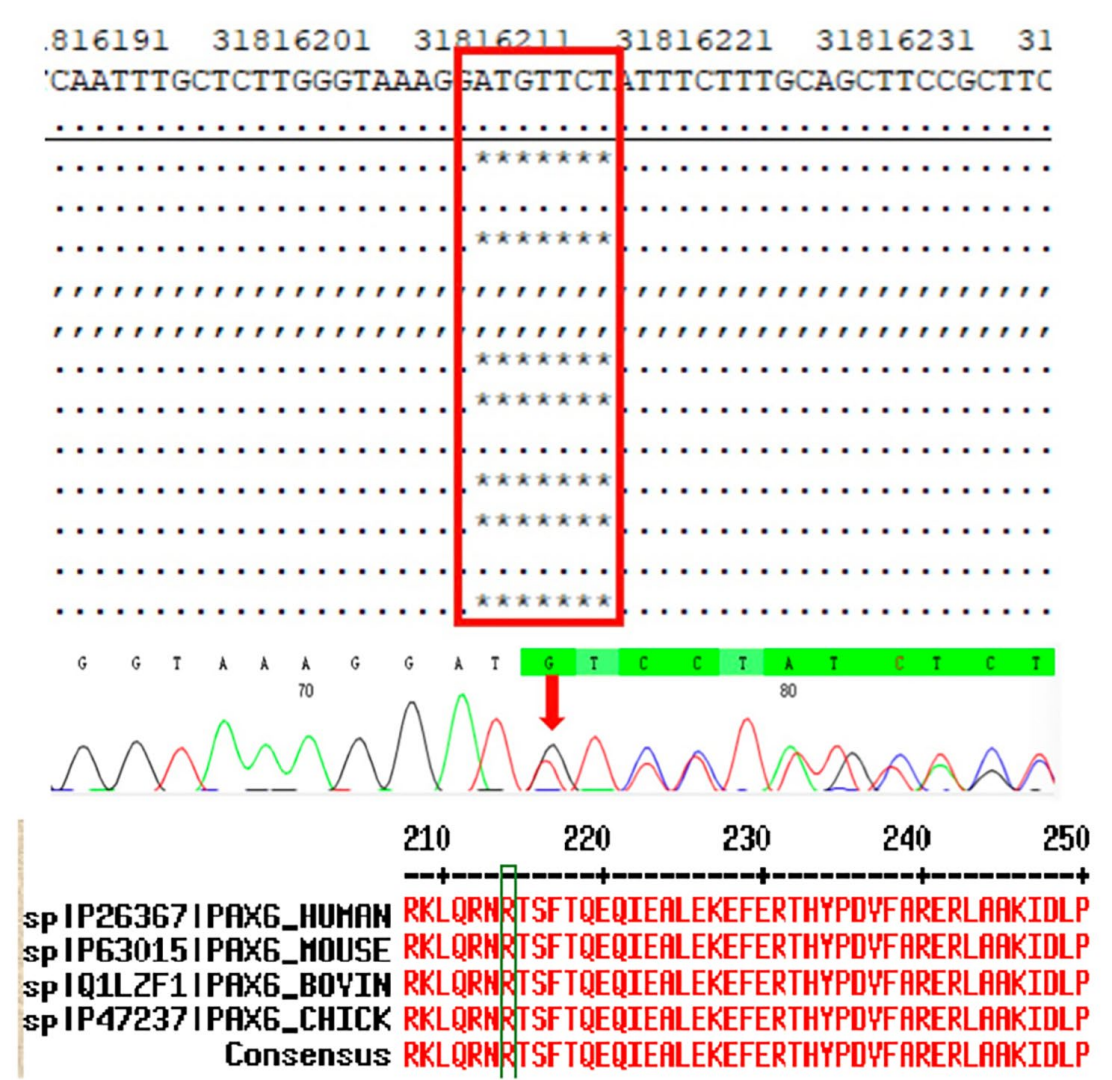
A Whole exome sequencing analysis was performed on the patient. A heterozygous variant c.640_646del (p. Arg214Profs*28) was identified. The c.640_646del variant, caused a frameshift starting with codon Arginine214, a position that was highly conserved among different species, as confirmed by proteomic conservation analysis

**Fig. 7:**
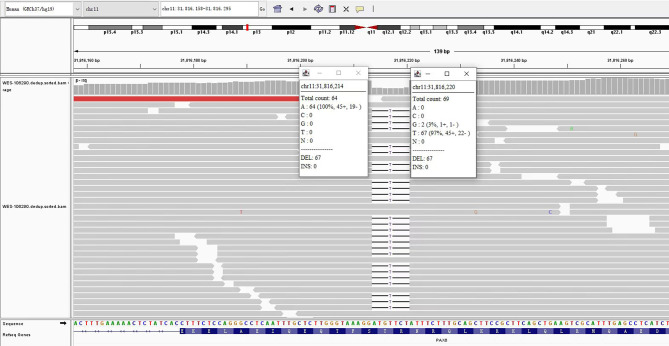
Minor Allele Frequency (MAF) analysis

Chromosomal analysis of peripheral lymphocytes revealed normal male karyotype (46, XY) at a band resolution of 400 bands per haploid genome. Whole exome sequencing analysis was performed on the patient, and a heterozygous variant c.640_646del (p. Arg214Profs*28) was found. The East Asian Population Database (ExAC_ EAS) the gnomAD database and prior reports of the frameshift variant were negative (PM2_Moderate). The *PAX6*(NM_000280.5)c.640_646del variant caused a frameshift starting with codon Arginine214, changing this amino acid to a proline residue, and creating a premature stop codon at position 28 of the new reading frame, denoted as p.Arg214Profs*28 (PP3_supporting)). This frameshift variant was located in the loss-of-function region (LOF) and affected the protein function (PVS1_Very Strong). Moreover, proteomic conservation analysis revealed that the amino acid at position 214 was highly conserved among different species (Fig. 6), indicating that the variant at this site is more likely to affect the structure and function of *PAX6* protein (PP3_Supporting). Since the parental samples were not available, the source of the variant was unknown. However, the combination analysis of MAF (Fig. 7) and Sanger sequencing further confirms that the c.640_646del variant is a true variant.

## Discussion

Congenital aniridia, affecting both the iris and other ocular structures such as the optic nerve, retina, cornea, and lens [19], is a complex disease caused by heterozygous mutations of the *PAX6* gene or associated regulatory regions, resulting in insufficient functional *PAX6* protein [20].

*PAX6* consists of 14 exons and is a member of the paired-box gene family located on chromosome 11p13. It encodes a transcriptional regulatory protein that recognizes target genes through paired DNA-binding domains, thereby regulating the transcription of downstream target genes [21, 22]. A hallmark feature of *PAX6* function is its gene dosage effect. Normal eye development depends on the presence of both copies of *PAX6*; therefore, loss of function in one of the two copies can lead to *PAX6* haploinsufficiency, causing aniridia, which can be found in 80–90% of patients [4]. This haploinsufficiency is caused by either chromosomal rearrangements or intragenic variations, or less commonly by mutations or deletions of nearby genes (TRIM44 and ELP4), which result in the silencing of *PAX6* [23, 24]. In 1991, *PAX6* was identified as the causative gene of congenital aniridia by positional cloning [21]. In their most recent update, which occurred in 2018, the online Human *PAX6* Mutation Databases (http://1sdb.hgu.mrc.ac.uk/home.php?select_db=PAX6) reported 491 mutations associated with aniridia. Since then, approximately 250 novel mutations described in the literature have been identified [1, 20, 25–34].

Missense mutations represented 11.7–17.5% of the sequence changes [35, 36]. Large heterozygous genomic deletions in the 11p13 region involving *PAX6* or its associated regulatory regions have also been frequently reported [36, 37]. The stop codon changed to a coding codon is defined as an “anti-termination” mutation and accounts for approximately 4% of the variation [38]. According to a review, 257 aniridia-associated mutations were classified as nonsense variation (38.9%), frame-shifting insertions or deletions (25.3%), splice variants (13.3%), missense variants (11.7%), in-frame insertions or deletions (6.2%), or run-on variants (4.7%) [39]. In another study on Chinese patients, these percentages were 33.3%, 19.0%, 14.3%, 9.5%, zero, and 9.5%, respectively, with an additional 14.3% gross deletions of the *PAX6* gene. Regions in exons 8, 9, 10, and 11 of the *PAX6* gene, which account for 21% of all mutations, are considered variant hotspots [35].

Nonsense variations may result in the synthesis of truncated proteins. However, these truncated proteins may be degraded in vivo through non-sense-mediated mRNA decay (NMD). NMD is an mRNA surveillance system found in higher eukaryotic cells. This mechanism typically degrades transcripts containing premature termination codons (PTCs), thereby limiting the synthesis of truncated proteins [40]. In most cases, this process can alters or affects the clinical phenotypes of certain genetic diseases. Non-sense variants present in different parts of the same gene can trigger or escape the NMD pathway [41]. However, when the PTC is within the final exon or the terminal 50 bp of the penultimate exon, the transcripts probably escape NMD and are translated into truncated proteins, resulting in a severe phenotype [42]. In this study, the patient carried a frameshift variant, and its stop codon was predicted to be upstream of exon 8, which might escape NMD and result in a severe clinical phenotype.

To date, there is no substantive evidence supporting concrete genotype-phenotype correlations [17, 43]. However, some connections were observed between the common mutations and clinical manifestations. Patients with typical aniridia, foveal hypoplasia, and nystagmus carry either an intragenic *PAX6* variant that induces the NMD process or a large deletion involving *PAX6* [1, 4]. Partial iris hypoplasia or a whole iris with an abnormal structure had either a run-on, missense, or splicing mutations that did not involve the NMD process [1]. C-terminal extension variants that lead to continuation of translation into the otherwise untranslated 3’ region of *PAX6* may generated extended *PAX6*proteins. Although phenotypes can vary widely even within families with the same variant, C-terminal extension variants are usually associated with severe phenotypes manifested as pronounced iris hypoplasia and severe visual impairment [44–46]. Chromosomal rearrangements (including deletions, duplications, translocations, and inversions) can cause isolated sporadic aniridia [47–49]. Chromosomal rearrangements disrupting the downstream cluster of ultraconserved transcriptional regulatory elements may affect *PAX6* expression and can also induce a typical aniridia phenotype without a sequence change in *PAX6* itself [24, 50]. Aniridia may also be caused by large genomic deletions encompassing *PAX6* and Wilms tumor 1 (*WT1)*, contiguous genes separated from each other by 700 kb, resulting in WAGR syndrome [49].

In this study, we report a heterozygous variant of exon8:c.640_646del:p.R214Pfs*28 of*PAX6* in a patient with congenital aniridia. To the best of our knowledge, this variant has not been reported previously. We described the patient’s clinical manifestations in detail and found it was consistent with previous research [4]. The patient showed complete iris aplasia, foveal hypoplasia, ectopic lentis, and retinal detachment in both eyes. Aniridia can increase the risks of retinal tears and detachments, even without a history of cataract surgery or other intraocular surgeries [51]. Interestingly, the retinal detachment in the patient’s right eye spontaneously reattached without treatment. During the patient’s first visit, the fundus examination revealed total retinal detachment of the right eye, and there was no significant change in retinal detachment during each follow-up examination. However, eight months after the initial diagnosis, during surgery in the right eye, we examined the fundus in detail; only a suspicious retinal tear was found behind the ora serrata at the 11 o’clock position, and retinal reattachment was achieved spontaneously. Spontaneous reattachment of rhegmatogenous retinal detachment (SRRRD) is a rare phenomenon, initially described in 1981 [52]. Since then, only a few SRRRD cases have been reported [53–58]. However, the mechanisms underlying SSRRD remain unclear. The suspected developmental mechanism involves the spontaneous relief of vitreoretinal traction with a complete posterior vitreous detachment (PVD) [56, 57]. The inflammatory response to retinal tears triggers microglial and Müller cell migration and massive proliferation at the vitreoretinal interface to establish a glial scar that closes retinal tears and may facilitate the development of SRRRD [57, 59, 60]. Chung et al. suggested that after the occurrence of PVD and retinal tears plugging, the retinal pigment epithelium removes residual subretinal fluid by active transport and/or by the Starling force (the balance between capillary pressure, interstitial pressure, and osmotic pressure) [53]. Congenital aniridia may be related to SRRRD, however, the specific cause of SRRRD in this patient is unclear. Complete or incomplete PVD and retinal tears plugging may have played a role in the SRRRD in this patient.

Although previous investigators have reported a few cases of aniridia with retinal detachment [51, 61, 62], there is currently no proven genotype-phenotype relationship between *PAX6*mutations and retinal detachment. Andersen et al. described an area of pathological vitreoretinal attachment, in which small retinal strands entered the anterior vitreous [63]. Jeseberg noted the occurrence of multiple, small, circumferentially distributed white lipid spots in the peripheral retina with aniridia [64]. However, in the present case, none of these manifestations were noted. Possible factors in the pathogenesis of retinal detachment in aniridia include prior surgery, vitreoretinal abnormalities related to aniridia, and buphthalmic ocular enlargement [51].

Although the patient presented in this study had only typical ocular manifestations, other patients with aniridia may have systemic diseases. Therefore, one of the primary goals of genetic evaluation in patients with aniridia is to exclude *PAX6* deletions that extend to the WT1 gene. Deletions in *WT1* put patients at risk of developing nephroblastoma or Wilms tumors [34].

## Conclusions

The present study reports a novel intragenetic deletion of the *PAX6* gene in a Chinese patient with congenital aniridia combined with ectopia lentis, cataracts, and retinal detachment. This result reveals additional genetic defects in the *PAX6* gene and broadens the *PAX6* pathogenic variants spectrum. As genetic analyses develop, a more detailed investigation of the clinical consequences of diverse *PAX6* variants is required.

## Data Availability

The datasets generated during the current study are available in the DDBJ BioSample repository, the sample of the patient can be obtained from the web link: https://ddbj.nig.ac.jp/resource/biosample/SAMD00567643.
